# Suppression of nbe-miR1919c-5p Expression in *Nicotiana benthamiana* Enhances Tobacco Curly Shoot Virus and Its Betasatellite Co-Infection

**DOI:** 10.3390/v12040392

**Published:** 2020-04-01

**Authors:** Jiang Du, Rui Wu, Zhuoying Liu, Miao Sun, Hussein Ghanem, Mingjun Li, Gentu Wu, Ling Qing

**Affiliations:** Chongqing Key Laboratory of Plant Disease Biology, College of Plant Protection, Southwest University, Chongqing 400716, China; dujiang20080803@163.com (J.D.); wr13598085291@163.com (R.W.); zz9629@email.swu.edu.cn (Z.L.); sunmiao4458@163.com (M.S.); h_aboelela@agr.asu.edu.eg (H.G.); lmj603@163.com (M.L.)

**Keywords:** tobacco curly shoot virus, betasatellite, *Nicotiana benthamiana*, nbe-miR1919c-5p, microRNA

## Abstract

MicroRNAs (miRNAs) are non-coding but functional RNA molecules of 21–25 nucleotides in length. MiRNAs play significant regulatory roles in diverse plant biological processes. In order to decipher the relationship between nbe-miR1919c-5p and the accumulations of tobacco curly shoot virus (TbCSV) and its betasatellite (TbCSB) DNAs, as well as viral symptom development, we investigated the function of nbe-miR1919c-5p during TbCSV and TbCSB co-infection in plants using a PVX-and a TRV-based short tandem target mimic (STTM) technology. Suppression of nbe-miR1919c-5p expression using these two technologies enhanced TbCSV and TbCSB co-infection-induced leaf curling symptoms in *Nicotiana benthamiana* plants. Furthermore, suppression of nbe-miR1919c-5p expression enhanced TbCSV and TbCSB DNA accumulations in the infected plants. Our results can advance our knowledge on the nbe-miR1919c-5p function during TbCSV and TbCSB co-infection.

## 1. Introduction

Plant microRNAs (miRNAs) are 21–25 nucleotides (nt) long and noncoding small RNAs, which can negatively regulate their target gene expression upon binding to mRNA sequences [1]. Numerous studies have also shown that through regulation of gene expression, miRNAs dictate many biological processes, including hormone homeostasis, leaf morphogenesis, root development, and plant responses to abiotic and/or biotic stresses [2,3,4,5,6,7].

Plant viruses are known to cause various disease symptoms: mosaic, leaf malformation, and stunting in infected plants. More recently, plant viruses have been shown to alter miRNAs expression in the infected plants [7,8,9,10,11,12,13,14,15]. Begomoviruses are single-stranded DNA viruses and often cause huge economical losses to many food crops [16]. Four distinct begomoviruses: African cassava mosaic virus (ACMV), cabbage leaf curl virus (CbLCuV), tomato yellow leaf curl virus (TYLCV), and cotton leaf curl multan virus plus its betasatellite (CLCuV/CLCuMB), have now been shown to regulate the expressions of ten different miRNAs in *N. benthamiana* plants [17]. Because the patterns of miRNA regulations by the four begomoviruses are different, it was speculated that different miRNAs can control different types and severity of disease symptoms in the infected plants [17].

The expression of miR159, miR319, and miR172 have been reported to delay the infection of tomato leaf curl New Delhi virus (ToLCNDV) in tomato plants cv Pusa Ruby. Similarly, the expressions of these miRNAs were found to be up-regulated in the ToLCNDV-infected tomato plants cv JK Asha or chilli pepper plants, resulting in leaf curling symptoms [18]. These results indicated that miR159, miR319, and miR172 may be involved in leaf symptom development. Based on these findings, Naqvi and others have suggested that the expression of miRNA(s) can be used as a molecular signature for ToLCNDV infection [18].

However, Romanel and colleagues have indicated that the current studies are not sufficient to link a particular miRNA to a specific viral symptom [19]. Till now, the function of nbe-miR1919c-5p has not been studied in the TbCSV and TbCSB (TbCSV/TbCSB) co-infected *N. benthamiana* plants. Our previous studies have shown that multiple miRNAs were differentially expressed in the TbCSV/TbCSB co-infected *N. benthamiana* plants [20]. In this study, we further explored the expression of miRNA in the TbCSV/TbCSB co-infected *N.benthamiana* plants and have identified a down-regulated miRNA, nbe-miR1919c-5p. Our results have confirmed that this miRNA is important for the formation of leaf curling symptom.

## 2. Materials and Methods

###  2.1. Gene Cloning and Vector Construction

To study the function of nbe-miR1919c-5p, the short tandem target mimic (STTM)-based system was used [21,22]. To construct the PVX-M1919 vector, a synthesized DNA containing a 48 base pair (bp) spacer sequence was double digested with the restriction enzymes *Cla*I and *Sal*I. The digested DNA product was inserted into the *Cla*I/*Sal*I site in a PVX vector, kindly provided by Prof. Jianping Chen (Ningbo University, Zhejiang, China), to produce pPVX-M1919. This plasmid was then digested with restriction enzyme *Pst*I, and the released DNA fragment was ligated into a pTRV-2 vector, also a gift from Prof. Jianping Chen, to produce pTRV2-M1919. To construct the pGD-OV1919 vector in which miR1919c-5p is over-expressed, the precursor of miR1919c-5p was PCR amplified using primers Pre1919-F and Pre1919-R. After double digestion with the restriction enzymes *Hind*III and *Sal*I, the PCR product was inserted into the *Hind*III/*Sal*I site of the pGD vector, kindly provided by Prof. Zaifeng Fan (China Agricultural University, Beijing, China). The constructs were sequenced and transformed, individually, into *Agrobacterium tumefaciens* strain GV3101. Primers used in this study are listed in Table S1.

### 2.2. Plant Growth, Virus Inoculation and Agro-infiltration

*N. benthamiana* plants were grown in a greenhouse set at 24–26 °C and a 16/8-h (light/dark) light cycle. Three-week-old plants were inoculated with the two viruses as previously reported [23]. Briefly, *A. tumefaciens* cultures carrying an infectious TbCSV (isolate Y35) clone or an infectious TbCSB clone were 1:1 (v/v) ratio mixed and then infiltrated into the *N. benthamiana* leaves using needleless syringes as described in [24]. For further analyses, A. tumefaciens cultures carrying pPVX-M1919, pTRV1, or pTRV2-M1919 were individually prepared. The single A. tumefaciens culture carrying pPVX-M1919 (refers to PVX-M1919) or the mixed *A. tumefaciens* culture harboring both pTRV1 and pTRV2-M1919 (refers to as TRV-M1919) were used to knock-down the expression of nbe-miR1919c-5p in N. benthamiana plants through agro-infiltration. To test the effect of nbe-miR1919c-5p on TbCSV/TbCSB infection, *N. benthamiana* plants were inoculated with PVX, PVX-M1919, TRV, or TRV-M1919 first through agro-infiltration. TbCSV/TbCSB was inoculated again onto PVX-M1919 or with a PVX empty vector (PVX-CK) treated plants after 7 days while for TRV-M1919-treated and TRV-CK plants after 14 days.

### 2.3. DNA Extraction and Virus DNA Accumulation Analysis

Total DNA was extracted from collected leaf samples using the cetyltrimethylammonium bromide (CTAB) method [25]. To determine the virus infection in the assayed plants, viral DNAs in young systemic leaves were detected through polymerase chain reaction (PCR) using TbCSV or TbCSB-specific primers [23]. To quantify viral DNA accumulation in the infected plants, we utilized a quantitative PCR (qPCR) methodology described before [26]. The resulting qPCR results were then calculated using the absolute quantification method as described in [27,28]. Briefly, full length TbCSV or TbCSB sequences were PCR amplified and cloned into the pEASY-T1 vector (TransGen Biotech, Beijing, China) to generate pT-TbCSV or pT-TbCSB plasmids. A 10-fold (10^9^ to 10 copies of plasmid DNA) serial-diluted pT-TbCSV or pT-TbCSB standard dilutions were prepared and used as controls. Specific TbCSV (TbCSV-qF/TbCSV-qR) or TbCSB (TbCSB-qF/TbCSB-qR) primers were designed to produce a 176 bp (TbCSV) or a 147 bp (TbCSB) amplicon. Each 20 µL qPCR reaction for TbCSV contained 10 µL NovoStart SYBR qPCR Super Mix Plus, 50 ng DNA template, 0.5 µL TbCSV-qF (10 µM), and 0.5 µL TbCSV-qR (10 µM) primers. The resulting standard curve for TbCSV appeared linear, had a coefficient of regression R^2^ = 0.990, and a calculated slope of −3.441. Each 20 µL qPCR reaction for TbCSB contained 10 µL NovoStart SYBR qPCR Super Mix Plus, 50 ng DNA template, 2.0 µL TbCSB-qF (10 µM), and 2.0 µL TbCSB-qR (10 µM) primers. The resulting standard curve for TbCSB also appeared linear, had a coefficient of regression R^2^ = 0.990, and a calculated slope of −3.39. Both graphs were generated using the Origin 9.0 software based on the lg (log10) value of TbCSV or TbCSB copy numbers in each sample. Each qPCR reaction was performed using three technical replicates and the results presented are the means from three individual experiments with 20 plants per experiment. Primers used in this study are all listed in Table S1.

### 2.4. RNA Extraction and Quantitative Reverse Transcription Polymerase Chain Reaction (qRT-PCR)

The procedure of qRT-PCR for nbe-miR1919c-5p was as previously reported [29]. For other qRT-PCR, total RNA was extracted from assayed plants with TRIzol as instructed (Invitrogen, California, USA). A specific stem-loop RT primer from the Prime Script RT reagent Kit (TAKARA Bio, Kusatsu, Shiga, Japan) was used for reverse transcriptions, and the RT products were used for the SYBR green kit (Novoprotein, Shanghai, China) based qPCR analyses using gene specific primers. The reaction conditions were 95 °C for 2 min; 40 cycles of 95 °C for 20 s and 60 °C for 20 s; and 72 °C for 30 s. The expression of *N. benthamiana Ubiquitin C* (UBC) gene was used as an internal control [30]. All reactions had three technical replicates and the data were analyzed using the 2^−ΔΔCT^ method. Primers used in this study are listed in Table S2.

### 2.5. Prediction and Quantification of Target Genes

Potential target genes of nbe-miR1919c-5p were predicted using the psRNATarget tool (http://plantgrn.noble.org/psRNATarget/, *Nicotiana benthamiana*, transcript, Niben101) [31]. To determine the expression of the target genes, we performed qRT-PCR using the NovoStart^®^ SYBR qPCR Super Mix Plus kit (Novoprotein) on a CFX 96 Real-Time System (Bio-Rad). The expression of *UBC* gene was used as an internal control [30]. All qRT-PCR reactions had three biological samples per treatment and three technical replicates per biological sample. The experiment was repeated three times. Primers used in this study are also listed in Table S2.

## 3. Results

### 3.1. Leaf Curling Symptoms Induced by TbCSV/TbCSB Co-Infection in N. benthamiana

**Figure 1:**
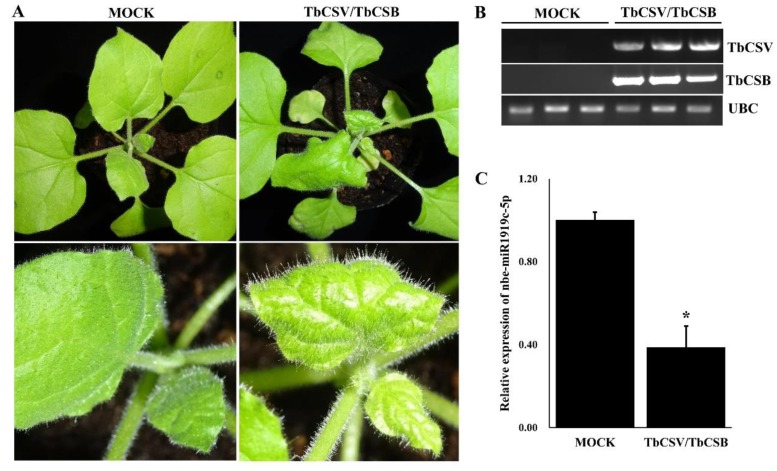
Co-infection of tobacco curly shoot virus (TbCSV) and its betasatellite (TbCSB) in *N. benthamiana* plants. (**A**) Leaf curling symptoms in a *N. benthamiana* plant co-inoculated with TbCSV and TbCSB. The plant inoculated with *Agrobacterium* cells without viral constructs (Mock) was used as control. The plants were photographed at 7 days post agro-infiltration (dpai). (**B**) Analysis of TbCSV and TbCSB DNA accumulations through PCR. The expression of *N. benthamiana Ubiquitin C* gene (*UBC*) was used as an internal control. (**C**) Analysis of nbe-miR1919c-5p expression in the mock- and the TbCSV/TbCSB-inoculated plants through qRT-PCR. Statistical differences between the treatments were determined by the Student’s *t*-test, * *p* < 0.05.

Twenty *N. benthamiana* plants were co-inoculated with TbCSV and TbCSB (refers to as TbCSV/TbCSB) through agro-infiltration. By 7 dpi, leaf curling symptoms were observed in the upper young developing leaves of the inoculated plants (Figure 1A). Results of PCR showed that TbCSV and TbCSB DNAs had accumulated in the symptomatic leaves (Figure 1B). In contrast, *N. benthamiana* plants inoculated with *Agrobacterium* cells without TbCSV and TbCSB infectious clones (mock-inoculated) did not show any virus-like symptoms and did not accumulate TbCSV or TbCSB DNA.

Our previous study had shown that the expression of nbe-miR1919c-5p was down-regulated in the TbCSV/TbCSB co-infected *N. benthamiana* plants. In this study, we analyzed the expression of nbe-miR1919c-5p again using qRT-PCR. Our result showed that the expression of nbe-miR1919c-5p in the infected plants was down-regulated by about 60% compared with the mock-inoculated plants (Figure 1C).

### 3.2. Suppression of Nbe-miR1919c-5p Expression Enhances Leaf Curling Symptoms Caused by TbCSV/TbCSB Co-Infection

**Figure 2:**
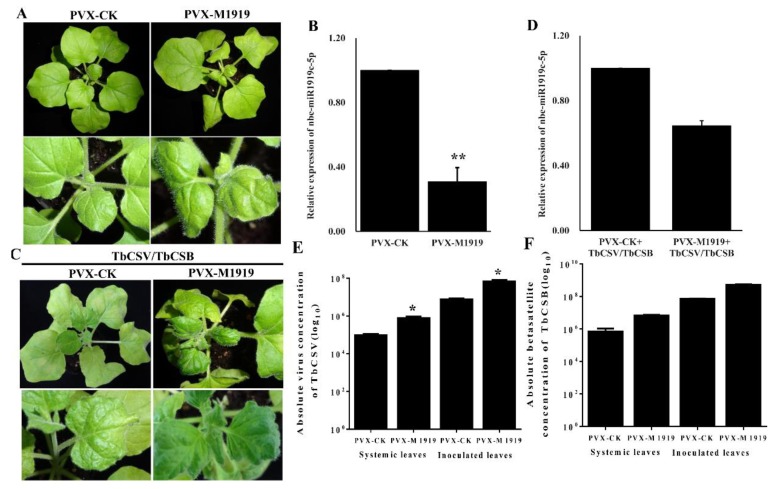
Suppression of nbe-miR1919c-5p expression enhances the leaf curling symptom induced by TbCSV/TbCSB infection and viral DNA accumulation. (**A**) A potato virus x (PVX)-based miRNA vector (PVX-M1919) was used to suppress the expression of nbe-miR1919c-5p. At 7 dpi, the PVX-M1919-inoculated plant showed strong leaf curling symptoms but not the control plant (PVX-CK). (**B**) qRT-PCR results showed that the expression of nbe-miR1919c-5p in the PVX-M1919-inoculated plants was significantly reduced compared with the control plants. (**C**) The PVX+TbCSV/TbCSB-inoculated and the PVX-M1919+TbCSV/TbCSB-inoculated plants were photographed 7 days after the second inoculation. Only the PVX-M1919+TbCSV/TbCSB-inoculated plants showed severe leaf curling symptoms. (**D**) qRT-PCR results showed that the expression of nbe-miR1919c-5p in the PVX-M1919+TbCSV/TbCSB-inoculated plants was significantly reduced. (**E**) Detections of TbCSV DNA copy numbers in the inoculated and the systemic leaves harvested from the PVX+TbCSV/TbCSB-inoculated plants or the PVX-M1919+TbCSV/TbCSB-inoculated plants. (**F**) Detection of TbCSB DNA copy numbers in the inoculated and the systemic leaves harvested from the PVX+TbCSV/TbCSB-inoculated plants or the PVX-M1919+TbCSV/TbCSB-inoculated plants. * *p* < 0.05; ** *p* < 0.01; determined by the Student’s *t*-test.

To explore the function of nbe-miR1919c-5p during TbCSV/TbCSB co-infection, a potato virus x (PVX)-based STTMs vector was used to suppress the expression of nbe-miR1919c-5p in *N. benthamiana* plants through agro-infiltration. PVX-M1919 and PVX were individually inoculated to 15 plants by agro-infiltration. At 7 days post agro-infiltration (dpi), the plants inoculated with PVX-M1919 showed clear leaf curling symptoms while the plants inoculated with PVX showed only mild PVX infection symptoms (Figure 2A). Results of qRT-PCR confirmed that the expression level of nbe-miR1919c-5p in the PVX-M1919-inoculated plants was significantly suppressed compared with the PVX-inoculated plants (Figure 2B).

At 7 days post PVX or PVX-M1919 inoculation, these plants were inoculated again with TbCSV and TbCSB. Seven days later, PVX and then TbCSV/TbCSB (PVX+TbCSV/TbCSB) inoculated plants showed leaf curling symptoms, similar to that caused by TbCSV/TbCSB co-infection. In contrast, the PVX-M1919 and then TbCSV/TbCSB (PVX-M1919+TbCSV/TbCSB) inoculated plants showed more severe leaf curling and malformation symptoms (Figure 2C). Analysis of these plants through qRT-PCR show that the expression level of nbe-miR1919c-5p in the PVX-M1919+TbCSV/TbCSB-inoculated plants was still strongly suppressed compared with the PVX+ TbCSV/TbCSB-inoculated plants (Figure 2D).

### 3.3. Suppression of Nbe-miR1919c-5p Expression Enhances TbCSV and TbCSB DNA Accumulation in N. benthamiana Plants

To determine the role of nbe-miR1919c-5p on TbCSV and TbCSB DNA accumulations in the infected plants, we analyzed the copy numbers of TbCSV and TbCSB DNAs in the inoculated or in the systemic leaves harvested from the PVX+TbCSV/TbCSB-inoculated or the PVX-M1919+TbCSV/TbCSB-inoculated plants through qPCR. All the leaf samples were collected at seven days post TbCSV/TbCSB inoculation. The results showed that the accumulations of TbCSV and TbCSB DNAs were significantly increased in both inoculated and systemic leaves harvested from the PVX-M1919+TbCSV/TbCSB-inoculated plants (Figure 2E,F).

**Figure 3:**
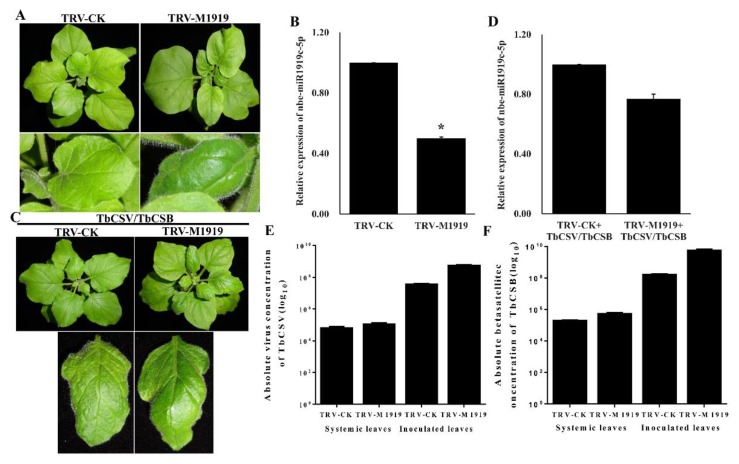
Suppression of nbe-miR1919c-5p expression using a TRV-based vector enhances leaf curling symptoms caused by TbCSV/TbCSB infection. (**A**) Plants inoculated with TRV or TRV-M1919 were photographed at 7 dpi. The TRV-M1919-inoculated plant showed stronger leaf curling symptoms compared with the TRV-inoculated control plants (TRV-CK). (**B**) Results of qRT-PCR showed that the expression of nbe-miR1919c-5p in the TRV-M1919-inoculated plants was significantly reduced compared with the control plants. (**C**) The TRV+TbCSV/TbCSB-inoculated and the TRV-M1919+TbCSV/TbCSB-inoculated plants were photographed 7 days after the second inoculation. The TRV-M1919+TbCSV/TbCSB-inoculated plants showed severe leaf curling symptoms. (**D**) Results of qRT-PCR showed that the expression of nbe-miR1919c-5p in the TRV-M1919+TbCSV/TbCSB-inoculated plants was significantly reduced compared with that in the TRV+TbCSV/TbCSB-inoculated plants. (**E**) Detection of TbCSV DNA copy number in the inoculated and the systemic leaves harvested from the TRV+TbCSV/TbCSB-inoculated or the TRV-M1919+TbCSV/TbCSB-inoculated plants. (**F**) Detection of TbCSB DNA copy number in the inoculated and the systemic leaves harvested from the TRV+TbCSV/TbCSB-inoculated or the TRV-M1919+TbCSV/TbCSB-inoculated plants. * *p* < 0.05 by the Student’s *t*-test.

The tobacco rattle virus (TRV)-based expression vector has been used as a tool to suppress the miRNA expressions in plants [32,33]. To further confirm the function of nbe-miR1919c-5p in TbCSV/TbCSB infection in plants, we inoculated the pTRV-M1919 vector or the pTRV vector to *N. benthamiana* leaves (15 plants per vector) through agro-infiltration. After 14 days, the TRV-M1919-inoculated plants showed clear leaf curling symptoms but not on the TRV-inoculated control plants (Figure 3A). Results of qRT-PCR confirmed that the expression of nbe-miR1919c-5p in the TRV-M1919-inoculated plants was suppressed by approximately 50% compared to the TRV-inoculated control plants (Figure 3B). We then co-inoculated TbCSV and TbCSB to the TRV-M1919- or the TRV-inoculated plants. At seven days post the second inoculation, the TRV and then TbCSV/TbCSB (TRV+TbCSV/TbCSB)-inoculated plants showed leaf curling symptoms, similar to that in the TbCSV/TbCSB-inoculated plants, while the TRV-M1919 and then TbCSV/TbCSB (TRV-M1919+TbCSV/TbCSB)-inoculated plants showed more severe leaf curling symptoms (Figure 3C). Results of qRT-PCR showed that at this time point, the expression of nbe-miR1919c-5p in the TRV-M1919+TbCSV/TbCSB-inoculated plants was still significantly reduced compared to the TRV+TbCSV/TbCSB-inoculated plants (Figure 3D). To check TbCSV and TbCSB DNA accumulations in the assayed plants, we harvested the inoculated and the young systemic leaves from individual assayed plants at seven days post the second inoculation, and analyzed them through qPCR. Results of qPCR showed that the levels of TbCSV and TbCSB DNAs in both inoculated and systemic leaves harvested from the TRV-M1919+TbCSV/TbCSB-inoculated plants were much higher compared with the TRV+TbCSV/TbCSB-inoculated plants (Figure 3E,F).

### 3.4. Over-Expression of Nbe-miR1919c-5p Reduced TbCSV and TbCSB DNA Accumulation in N. benthamiana Plants

**Figure 4:**
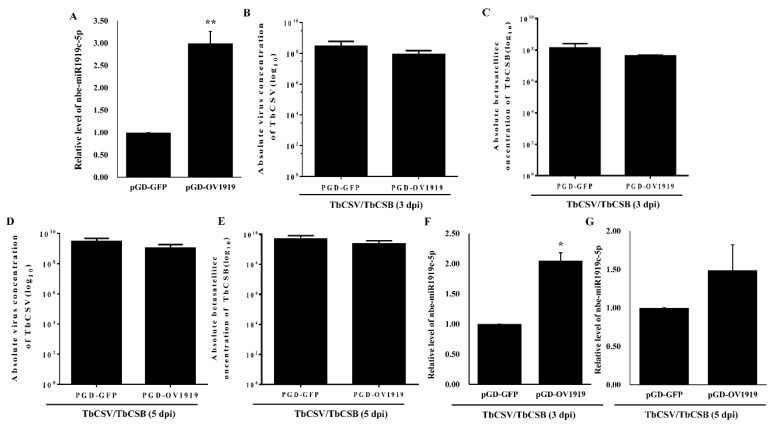
The accumulations of TbCSV and TbCSB DNAs in the *N. benthamiana* leaves transiently over-expressed nbe-miR1919c-5p. (**A**) Result of qRT-PCR showed that the expression of nbe-miR1919c-5p in the pGD-OV1919-inoculated plants was significantly increased compared with the control plants (pGD-GFP). (**B**) Detection of TbCSV DNA copy number in the inoculated leaves harvested from the pGD-OV1919+TbCSV/TbCSB-inoculated or the pGD-GFP+TbCSV/TbCSB-inoculated plants at 3 dpi. (**C**) Detection of TbCSB DNA copy number in the inoculated leaves harvested from the pGD-OV1919+TbCSV/TbCSB-inoculated or the pGD-GFP+TbCSV/TbCSB-inoculated plants at 3 dpi. (**D**) Detection of TbCSV DNA copy number in the inoculated leaves harvested from the pGD-OV1919+TbCSV/TbCSB-inoculated or the pGD-GFP+TbCSV/TbCSB-inoculated plants at 5 dpi. (**E**) Detection of TbCSB DNA copy number in the inoculated leaves harvested from the pGD-OV1919+TbCSV/TbCSB-inoculated or the pGD-GFP+TbCSV/TbCSB-inoculated plants at 5 dpi. (**F**) Results of qRT-PCR showed that the expression of nbe-miR1919c-5p in the pGD-OV1919+TbCSV/TbCSB-inoculated plants was significantly increased compared with that in pGD-GFP+TbCSV/TbCSB-inoculated plants at 3 dpi. (**G**) Results of qRT-PCR showed that the expression of nbe-miR1919c-5p in the pGD-OV1919+TbCSV/TbCSB-inoculated plants was increased compared with that in pGD-GFP+TbCSV/TbCSB-inoculated plants at 5 dpi. * *p* < 0.05; ** *p* < 0.01; determined by the Student’s *t*-test.

In order to explore the function of nbe-miR1919c-5p deeply, a pGD vector was used to transiently over-express the expression of nbe-miR1919c-5p in *N. benthamiana* plants through agro-infiltration. pGD-OV1919 and pGD-GFP were individually inoculated to 15 plants by agro-infiltration. At 2 dpi, the result showed that the expression of nbe-miR1919c-5p was up-regulated significantly by 3-fold, compared with that in the pGD-GFP-inoculated control plants (Figure 4A). Also at 2 dpi, the infiltrated leaves were inoculated again with TbCSV and TbCSB. After 3 and 5 days post the second inoculation, we analyzed the copy numbers of TbCSV and TbCSB DNAs in the inoculated leaves harvested from the pGD-GFP+TbCSV/TbCSB-inoculated or the pGD-OV1919+TbCSV/TbCSB-inoculated plants through qPCR, respectively. The results showed that the accumulations of TbCSV and TbCSB DNAs were reduced in inoculated leaves harvested from the pGD-OV1919+TbCSV/TbCSB-inoculated plants at 3 and 5 dpi, respectively (Figure 4B–E). At 3 and 5 dpi, the expression of nbe-miR1919c-5p in the pGD-OV1919+TbCSV/TbCSB-inoculated plants was still over-expressed compared with the pGD-GFP+TbCSV/TbCSB-inoculated plants, respectively (Figure 4F,G).

### 3.5. Quantification of Nbe-miR1919c-5p Target Gene Expression

**Figure 5:**
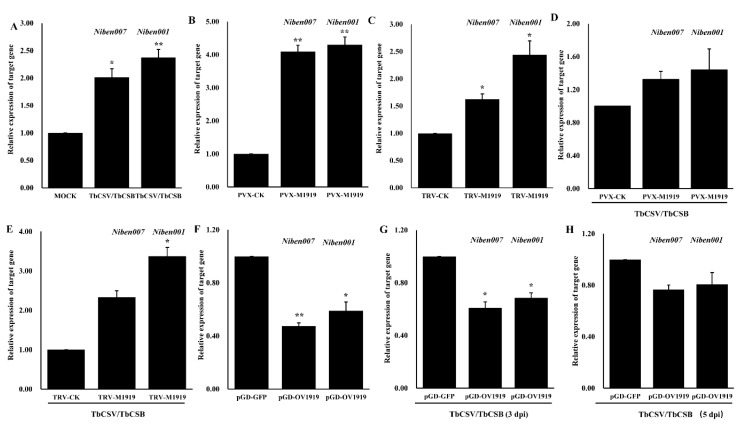
Analyses of the expression of nbe-miR1919c-5p target genes (*Niben001* and *Niben007*) through qRT-PCR. (**A**) At seven days post TbCSV/TbCSB co-inoculation, the assayed plants were sampled and analyzed for the expression of *Niben001* and *Niben007* through qRT-PCR. *N. benthamiana* plants inoculated with *Agrobacterium* cells without virus infectious clones (Mock) were used as controls. (**B**) Detection of *Niben001* and *Niben007* expression in the PVX-inoculated or the PVX-M1919-inoculated plants through qRT-PCR. (**C**) Detection of *Niben001* and *Niben007* expressions in the TRV-inoculated or the TRV-M1919-inoculated plants through qRT-PCR. (**D**) Detection of *Niben001* and *Niben007* expressions in the PVX+TbCSV/TbCSB-inoculated or the PVX-M1919+TbCSV/TbCSB-inoculated plants through qRT-PCR. (**E**) Detection of *Niben001* and *Niben007* expression in the TRV+TbCSV/TbCSB-inoculated or the TRV-M1919+TbCSV/TbCSB-inoculated plants through qRT-PCR. (**F**) Detection of *Niben001* and *Niben007* expression in the pGD-OV1919-inoculated or the pGD-GFP-inoculated plants through qRT-PCR. (**G**) Detection of *Niben001* and *Niben007* expressions in the pGD-OV1919+TbCSV/TbCSB-inoculated or the pGD-GFP-M1919+TbCSV/TbCSB-inoculated plants through qRT-PCR at 3 dpi. (**H**) Detection of *Niben001* and *Niben007* expressions in the pGD-OV1919+TbCSV/TbCSB-inoculated or the pGD-GFP-M1919+TbCSV/TbCSB-inoculated plants through qRT-PCR at 5 dpi. * *p* < 0.05, ** *p* < 0.01; determined by the Student’s *t*-test.

To further investigate how nbe-miR1919c-5p regulates TbCSV and TbCSB co-infection in plants, we searched the psRNATarget website (http://plantgrn.noble.org/psRNATarget/) to find potential *N. benthamiana* genes targeted by nbe-miR1919c-5p (Table 1). We then designed specific PCR primers (Table S2) for these target genes for qRT-PCR. The results showed that at 7 days post TbCSV and TbCSB co-inoculation, the expression of *Niben001* and *Niben007* were up-regulated significantly by 2.5 and 2-fold, respectively, compared with that in the mock-inoculated control plants (Figure 5A). We then analyzed the expression of *Niben001* and *Niben007* in the PVX-M1919- or the PVX-inoculated plants by qRT-PCR. The results showed that the expression of *Niben001* and *Niben007* in the PVX-M1919-inoculated plants were increased significantly by about 4.2 and 4 fold, respectively, compared with the PVX-inoculated control plants (Figure 5B). In a separate assay, we have found that the expression of *Niben001* and *Niben007* in the TRV-M1919-inoculated plants were increased significantly by about 2.5 and 1.5 fold, respectively, compared with the TRV-inoculated control plants (Figure 5C). In addition, we have found that the expression of *Niben001* and *Niben007* in the PVX-M1919+TbCSV/TbCSB-inoculated plants were increased by about 1.5 and 1.4 fold, respectively, compared with the PVX+TbCSV/TbCSB-inoculated plants (Figure 5D). The expression of *Niben001* and *Niben007* in the TRV-M1919+TbCSV/TbCSB-inoculated plants were increased by about 3.5 and 2.5-fold, respectively, compared with the TRV+TbCSV/TbCSB-inoculated plants (Figure 5E). Our results showed that at 2 days post pGD-OV1919 inoculation, the expression of *Niben001* and *Niben007* were down-regulated significantly by 50% and 40%, respectively, compared with that in the pGD-GFP-inoculated control plants (Figure 5F). We also analyzed the expression of *Niben001* and *Niben007* in the pGD-OV1919+TbCSV/TbCSB or the pGD-GFP+TbCSV/TbCSB-inoculated plants by qRT-PCR. The results showed that the expression of *Niben001* and *Niben007* in the pGD-OV1919+TbCSV/TbCSB-inoculated plants were both reduced at 3 and 5 days post inoculation compared with the pGD-GFP+TbCSV/TbCSB-inoculated control plants, respectively (Figure 5G,H).

## 4. Discussion

It has been shown that miRNAs play important roles in plant resistance against pathogens. The expressions of miRNAs are often changed after plant virus infections and several miRNAs have been determined to regulate virus symptoms development. For example, several begomoviruse infections have been reported to alter miR159, miR160, miR164, miR165, miR166, miR167, miR168, miR169, and miR170 expression [17]. The change of certain miRNA expressions can result in downward or upward leaf curling, vein yellowing, vein swelling, and plant stunting. In our previous report, we have shown, through small RNA sequencing, that the expression of about 13 known miRNAs, including miR156a, miR160a, miR169a, miR171b, miR395a, miR482a, and miR1919c-5p, were differentially regulated in the TbCSV/TbCSB -infected *N. benthamiana* plants [20]. We have speculated that some of these differentially regulated miRNAs may control virus symptoms development.

To date, miR1919 has only been found in *Solanaceae* species through searching the miRbase database (http://www.mirbase.org). Baksa and colleagues have reported that nbe-miR1919 is highly expressed in *N. benthamiana* leaves based on their high-throughput sequencing result [34]. Feng and others have also found that the expression of sly-miR1919 is down-regulated in tomato plants infected with cucumber mosaic virus (CMV), based on their deep sequencing results [4]. In our previous study, we have reported that the expression of nbe-miR1919c-5p in the *N. benthamiana* plants co-infected with TbCSV and TbCSB was altered [20]. In this study, we have found that the expression of nbe-miR1919c-5p in the *N. benthamiana* plants at 3, 5, 7, 9, 10 days post TbCSV/TbCSB inoculation was down-regulated compared with mock-inoculated plants (Figure S1). Because the expression of nbe-miR1919c-5p was suppressed in the TbCSV/TbCSB-infected plants, we consider that the nbe-miR1919c-5p may play a negative role during TbCSV and TbCSB DNA accumulations.

It has been demonstrated that the reduction of osa-miR171b expression in rice plants infected with rice stripe virus (RSV) causes RSV symptoms formation [35]. Wang and others have reported that the suppression of nbe-miR166h-p5 expression in plants attenuates leaf yellowing symptoms and reduces virus accumulation [36]. Zhang and others have revealed that the induction of miR319 expression in rice by rice ragged stunt virus (RRSV) infection suppresses rice JA-mediated defense to facilitate virus infection and symptom development [8]. In this report, we have shown that the suppression of nbe-miR1919c-5p in *N. benthamiana* plants increases TbCSV and TbCSB DNA accumulations, and enhances disease symptoms (Figure 2A and Figure 3A). Also in this study, we constructed an over-expression vector for nbe-miR1919 (pGD-OV1919) and agro-infiltrated this vector to *N. benthamiana* leaves. At 2 dpi, the infiltrated leaves were inoculated again with TbCSV and TbCSB. At 3 and 5 days post the second inoculation, the inoculated leaves were analyzed for TbCSV and TbCSB DNA accumulations through qPCR. Our result showed that the levels of TbCSV and TbCSB DNAs in the leaves transiently over-expressing nbe-miR1919c-5p were decreased (Figure 4), indicating an association between nbe-miR1919c-5p and plant resistance against TbCSV/TbCSB infection.

We then predicted and tested the expressions of two nbe-miR1919c-5p target genes after various treatments. Although our results showed that the expression of these two genes was regulated by TbCSV/TbCSB infection, future studies of these two genes need to be investigated. In particular, stable over-expression of these two target genes in plants may provide some useful information on the relationship between nbemiR1919c-5p and the two target genes and/or on TbCSV/TbCSB infection.

In this study, we utilized a miRNA target mimic (TM) technology to investigate the function of miR1919c-5p during TbCSV/TbCSB infection in plant. Zhao and others have reported that the PVX-based STTM system for miR165/166 or 159 can be used to induce corresponding phenotypes in the inoculated plants, and the PVX-based STTM system is more efficient than the TRV-based STTM system for miRNA expressions in the leaves of *N. benthamiana* [32]. Wang and others have found that the suppression of nbe-miR166h-p5 expression in plants causes darker green leaves mainly because of the increase in chlorophyll content [36]. In this study, we used both PVX- and TRV-based STTM system to suppress nbe-miR1919c-5p expression in *N. benthamiana* plants and obtained leaf curling symptoms in the assayed plants. We have found that the PVX-based STTM system appears to be more efficient than the TRV-based STTM system for the suppression of miR1919c-5p expression in *N. benthamiana*.

## 5. Conclusions

In this study, we have analyzed the effects of nbe-miR1919c-5p on TbCSV/TbCSB infection-induced symptom development and viral DNA accumulation. We have shown that suppression of nbe-miR1919c-5p expression in plants can enhance leaf curling symptoms and the accumulations of TbCSV and TbCSB DNAs in the infected *N. benthamiana* plants. The effect of nbe-miR1919c-5p on TbCSV/TbCSB infection in plants have not been reported previously.

## Figures and Tables

**Table 1:** Predictions of nbe-miR1919c-5p targeted *N. benthamiana* genes.

miRNA	Target Gene	Expectation	UPE	miRNA Start	miRNA End	Target Start	Target End	miRNA Aligned Fragment	Target Aligned Fragment	Inhibition	Multiplicity
Nbe-miR1919c-5p	Niben101Scf04663g00007	2.0	15.475	1	21	254	274	UGUCGCAGAUGACUUUCGCCC	AUGCGAAAGUCAUCUGCGACA	Cleavage	1
Nbe-miR1919c-5p	Niben101Scf02655g01001	2.0	16.74	1	21	485	505	UGUCGCAGAUGACUUUCGCCC	AUGCGAAAGUCAUCUGCGACA	Cleavage	1

## Supplementary Materials

The following are available online at https://www.mdpi.com/1999-4915/12/4/392/s1, Table S1. PCR primers used for TbCSV and TbCSB detections and plasmids construction. Table S2. qRT-PCR primers used for miRNA and mRNAs detections. Figure S1. The expression level of nbe-miR1919c-5p in TbCSV and TbCSB co-infected *N. benthamiana* plants.
