# Supplementary material for: Suppression of nbe-miR1919c-5p Expression in Nicotiana benthamiana Enhances Tobacco Curly Shoot Virus and Its Betasatellite Co-Infection
Source: Viruses. 2020 Apr 1;12(4):392. doi: 10.3390/v12040392 (PMC7232422; doi:10.3390/v12040392)
Supplement: Supplementary file 1 [file viruses-12-00392-s001.zip › viruses-732941-for conversion/Table S1 PCR primers used for TbCSV and TbCSB detections..pdf]

Table S1 PCR primers used for TbCSV and TbCSB detections.

mRNA primers	Primer sequence (5'-3')	Brief description	
TbCSV-F	ATGCCTCAGCCAAGAAAACTTTT	Amplify *AC1* (1086 bp) for	[1]
TbCSV-R	TCAACACGACGACGTCTGTTCCC	detecting TbCSV	
TbCSB-F	ATGACAATTAAATACAACAACAAG	Amplify *βC1* (357 bp) for	
TbCSB-R	TCATACATTAGCTATTGTCCC	detecting TbCSB	
Pre1919-F	CGC*GGATCC*TCCAAAAATCGAATCGTTCC	Amplify the precursor of	
Pre1919-R	ACGC*GTCGAC*GATGAAAACGCCCCCAATAA	miR1919c-5p (200 bp)	

1. Li, K.; Wu, G.; Li, M.; Ma, M.; Du, J.; Sun, M.; Sun, X.; Qing, L., Transcriptome analysis of Nicotiana benthamiana infected by Tobacco curly shoot virus. *Virol J* **2018,** 15, (1), 138.
