# Supplementary material for: Suppression of nbe-miR1919c-5p Expression in Nicotiana benthamiana Enhances Tobacco Curly Shoot Virus and Its Betasatellite Co-Infection
Source: Viruses. 2020 Apr 1;12(4):392. doi: 10.3390/v12040392 (PMC7232422; doi:10.3390/v12040392)
Supplement: Supplementary file 1 [file viruses-12-00392-s001.zip › viruses-732941-for conversion/Table S2 qRT-PCR primers used for miRNA and mRNAs detections..pdf]

Table S2 qRT-PCR primers used for miRNA and mRNAs detections.

primers	Primer sequence (5’-3’)	
miR1919c-5p-RT	GTTGGCTCTGGTGCAGGGTCCGAGGTATTCGCACCAGAGCCA ACGGGCGAA	
miR1919c-5p-qF	CGCGCAGTGTCGCAGATGACT	
Universal-qR	GGTGCAGGGTCCGAGGTAT	
TbCSV-qF	CGCCGCCGTCTCAACTTCG	
TbCSV-qR	GACTGGACCTTACATGGACCTTCAC	
TbCSB-qF	CGTTGATGTTAAGTTGAAGGAGGAC	
TbCSB-qR	TCTAAGTTATTGAAATTGAATGGTGGTATG	
Niben007-qF	TGGAGTTTATTCTGGAAATTAC	
Niben007-qR	TAGAAAAAGAGTGAACTTGAATC	
Niben001-qF	ACTTGCTTCCACTCCACTTGAT	
Niben001-qR	CAGAATAGAAAAAGAGCGAACT	
UBC-qF	TTTCGGTCCTGATGATACTCCC	[1]
UBC-qR	CACAGAGCAAAGACTGGATTGA	

1. He, Q.; Peng, J.; Yan, F.; Lin, L.; Lu, Y.; Zheng, H.; Chen, H.; Chen, J., Intron retention and 3′-UTR analysis ofArabidopsisDicer-like 2 transcripts. 39, (3), 3271-3280.
