# Supplementary figures and images for: Suppression of nbe-miR1919c-5p Expression in Nicotiana benthamiana Enhances Tobacco Curly Shoot Virus and Its Betasatellite Co-Infection
Source: Viruses. 2020 Apr 1;12(4):392. doi: 10.3390/v12040392 (PMC7232422; doi:10.3390/v12040392)

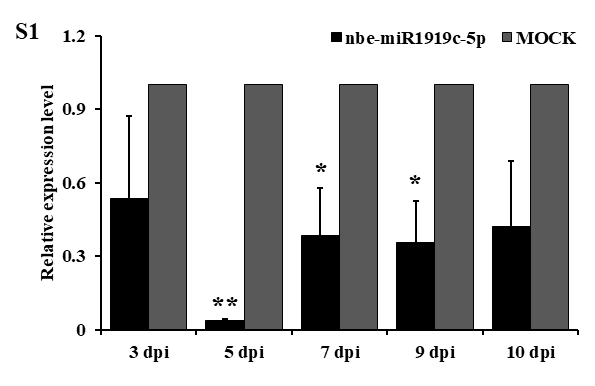

Supplement: Supplementary file 1 [file viruses-12-00392-s001.zip › viruses-732941-for conversion/Figure S1 The expression level of nbe-miR1919c-5p when N. benthamiana plants infected with TbCSVTbCSB.tif]
